# Unexplained Deaths and Critical Illnesses of Suspected Infectious Cause, Taiwan, 2000–2005

**DOI:** 10.3201/eid1410.061587

**Published:** 2008-10

**Authors:** Tsung-Hsi Wang, Kuo-Chen Wei, Donald Dah-Shyong Jiang, Chan-Hsian Chiu, Shan-Chwen Chang, Jung-Der Wang

**Affiliations:** Taiwan Centers for Disease Control, Taipei, Taiwan (T.-H. Wang; D.D. Jiang, C.-H. Chiu); Chang Gung University and Chang Gung Memorial Hospital, Taoyuan, Taiwan (K.-C. Wei); College of Medicine of the National Taiwan University, Taipei (S.-C. Chang, J.-D. Wang); College of Public Health of the National Taiwan University, Taipei (J.-D. Wang)

**Keywords:** Cause of death, autopsy, Taiwan, infection, bacteria, viruses, dispatch

## Abstract

We report 5 years’ surveillance data from the Taiwan Centers for Disease Control on unexplained deaths and critical illnesses suspected of being caused by infection. A total of 130 cases were reported; the incidence rate was 0.12 per 100,000 person-years; and infectious causes were identified for 81 cases (62%).

In 2003, the outbreak of severe acute respiratory syndrome (SARS) demonstrated that the world has become a global village in which human risk for exposure to different kinds of biological hazards is increased through frequent travel and commercial activities (*1*–*5*). Historically, emerging diseases occur abruptly in outbreaks of unknown cause. Although various efforts have been proposed and conducted to analyze secondary data periodically (*6*–*9*), they generally provide information for the less urgent decision making in health policy and may not be in time for infectious disease control. Thus, a task force is needed to provide timely and accurate diagnosis for early control of any potential epidemic infection, especially in a newly developed country like Taiwan, where the healthcare resources may not be evenly distributed and autopsy for diagnosis is not widely accepted culturally.

In 2000, the Taiwan Centers for Disease Control collaborated with academic institutions, medical examiners, local health authorities, and experts from different fields to establish a nationwide surveillance center for outbreak and unexplained death investigation due to unknown infectious causes (COUNEX) (Figure). This effort was to build Taiwan’s capacity for detecting and responding to uncommon and unrecognized pathogens, which was conceptually the same as that of the study of Hajjeh et al. (*10*). We defined the surveillance case-patient as a previously healthy resident who died or was admitted to a hospital with a life-threatening illness possibly caused by infection of unidentified etiology. Usually the death occurred within 3 days of the patient’s admission. Patients were excluded if the cause of death was noninfectious. A life-threatening illness was defined as any illness requiring admission to an intensive care unit or report as being critical. An infectious disease is generally suspected if the case-patient has >1 of the characteristics such as fever, leukocytosis, histopathologic evidence of an acute infectious process or more specific symptom patterns, or infection precipitating adult respiratory distress syndrome, renal failure, or sepsis.

**Figure:**
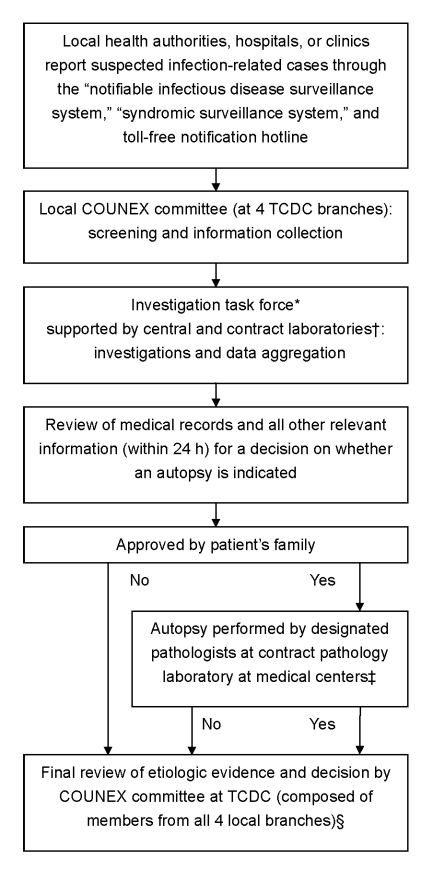
Flow of information and decision making for reported cases of unexplained death or critical illness. *If unexplained infectious causes were suspected, COUNEX mobilized an investigation team including experts, field epidemiology training program members, public health workers from the local branch of Taiwan Centers for Disease Control (TCDC), and public health authorities to proceed with further field investigation. TCDC was in charge of the investigation. †Cases were categorized into >1 of the following clinical syndromes: acute neurologic (encephalitis, meningitis), acute respiratory (pneumonia), acute hemorrhagic, acute diarrhea, acute jaundice (hepatitis), acute heart (myocarditis, pericarditis, endocarditis), and acute kidney-related. For every reported case, COUNEX investigators usually selected diagnostic tests relevant to a particular syndrome (www.cdc.gov.tw). Additional tests were prescribed if needed. The hospital laboratories were requested to save all remaining clinical specimens, including biopsy specimens, obtained from clinical management and send them to our reference laboratories, if indicated. ‡If an autopsy was performed, whenever possible tissue specimens were examined by pathologists of TCDC-designated medical centers and the Forensic Department of the Ministry of Justice to ensure the accuracy of the final diagnosis. Specimens were also sent for microbiologic cultures and tests as well as toxicologic examination for trace toxic chemicals, if needed. §All laboratory results and clinical, epidemiologic, and pathologic data were sent to the expert committee to determine if the etiologic agent could fully or most likely explain the disease. Otherwise, cases were categorized as unexplained. In general, histopathogic examination was the major evidence for determining cause. If case-patients could not be autopsied within 36 hours of death, laboratory results would be the most useful information for identification of cause of death.

A total of 130 cases were reported during 2000–2005, for an annual average rate of 0.12 cases per 100,000 persons. The annual incidence rates varied by year and among 4 branches of Taiwan Centers for Disease Control (Table). The highest rate was in the eastern branch, where surveillance was conducted in a well-defined population of ≈596,119 persons. Ninety-five (73%) of the case-patients died. For 47 (49%) of those who died, an autopsy was performed, a rate much higher than the national autopsy rate of <11% (*12*). The mean age of case-patients was 33.8 years. The incidence rates varied by age group; it was highest in those 85–89 years of age, followed by those <1–4 years, and then 65–69 years, with 0.48, 0.30, and 0.23 per 100,000 person-years, respectively. Men had a higher incidence rate than women (0.16 vs. 0.10 per 100,000 person-years).

**Table:** Incidence rate of case-patients detected by surveillance and proportions of deaths, possible infectious causes, and autopsy, Taiwan, August 2000–March 2005

Category	TCDC branch*				
	Total	Northern	Middle	Southern	Eastern
Incidence/100,000 person-years†	0.12	0.09	0.16	0.09	0.64
Proportion of deaths among all case-patients, %	73	75	64	79	83
Proportion of infectious causes identified, %	65	63	68	68	39
Viral agents among infection cases, %	42	56	30	37	57
Bacterial agents among infection cases, %	46	36	57	47	36
*Rickettsia* spp. among infection cases, %	4	4	3	0	14
Proportion of causes remaining unknown, %	23	25	16	29	25
Autopsy rate among patients who died, %	49	53	43	41	67

*TCDC, Taiwan Centers for Disease Control. †Denominators for the population under surveillance, obtained from the 2002 intercensus (*11*) and approximately the midpoint of this study period, included all people in the age groups under surveillance at the various sites and were used to calculate the incidence rate.

Approximately 10% of 130 case-patients and 16% of 81 patients with cases of infection had a history of animal contact; 9% of 130 case-patients and 10% of infection case-patients had a history of travel outside Taiwan within the previous 3 months. The most common initial syndromes were acute respiratory (59%), acute neurologic (22%), and acute diarrhea-related syndrome (13%). Initially, 8 patients had acute heart-related syndrome, and 11 had acute kidney-related syndrome; both of these syndromes had a 100% case-fatality rate.

The Appendix Table lists all the infectious pathogens and noninfectious causes identified among 95 fatal cases. One third were related to bacterial infection and one fourth to viral infection; 22 remained unclassified. The proportion of explained cases was lower among patients who survived (74%) than that among patients who died (77%). The proportion of explained cases was also higher for patients who underwent autopsy (83%) than for nonautopsied patients (71%) but not statistically significantly so. Explained cases were similar to unexplained cases in terms of patient age and interval between dates of disease onset and report (median 7.2 and 6.8 days, for explained and unexplained cases, respectively). Although the overall case-fatality rate was 73%, patients were more likely to die if they had multiple organ system involvement.

We have established the infrastructure needed to detect critical and fatal cases of unknown causes; such a surveillance system is essential to identify early potential infectious threats in a period of globalization and increasing travel between countries. The contributions of our surveillance system are demonstrated by early detection and control of at least 3 outbreaks of serious viral diseases: hantavirus pulmonary syndrome, rabies, and SARS.

In 2001, a family cluster occurred in Huanlian city; dyspnea, cough, leukopenia, and pulmonary edema developed in both parents, who died. Their 16-year-old daughter was also ill, but she survived. COUNEX quickly intervened, and hantavirus pulmonary syndrome was confirmed by positive serologic test results, which led to an early control of local rodents and spread of the disease.

Taiwan has been free of human and animal rabies since 1961. However in 2002, a 45-year-old woman from mainland People’s Republic of China was admitted to a hospital because of difficulty in swallowing, fear of wind (aerophobia), and numbness of the arms. Her condition was reported to the surveillance system as suspected rabies. Our personnel quickly confirmed the diagnosis by reverse transcription–PCR and DNA sequence analysis of the samples from cerebrospinal fluid, saliva, and trachea while the patient was still alive (*13*). The patient had been bitten by a domesticated dog in mainland China 2 months earlier.

During the SARS outbreak in 2003, the surveillance system received reports of 6 cases; autopsies were performed on 3 patients. As a result, the correlation between clinical course and pulmonary pathology at different stages of the disease was possible, and corroborative evidence for control measures was provided (*14*).

Had the surveillance system for unexplained death and critical illness not functioned normally during these 3 outbreaks, more people in Taiwan would have been ill and died from the diseases because of the high population density on this island. This system was particularly useful for infection control at remote regions with limited resources. Most physicians in the rural eastern part of the country have less access to consultation and referral to other specialties in medical centers and teaching hospitals. Thus, they rely more on this kind of surveillance system for early detection of potential infectious threats. This was especially important for acute unexpected deaths, as was demonstrated by a higher incidence and autopsy rates in eastern Taiwan.

Throughout this project, we have increased the autopsy rate and established a population-based bank of specimens for future research. This collection could provide a better opportunity for corroboration or refutation of any previous diagnosis of infectious disease. This improved decision making in regard to control of infections was demonstrated in November 2003, when influenza virus (H5N1) was diagnosed in a patient who had a previous misdiagnosis of SARS (*15*).

Because emerging and reemerging infectious diseases may quickly travel between different countries, the system is becoming more crucial for early detection and control of potential health hazards. The system depends on close cooperation among different disciplines and staff from different agencies. Thus, education, empowerment, and good feedback incentives should be continually offered to keep this system sustainable.

## Supplementary Material

Appendix Table Frequency distributions of identified pathogens for acute cases with unexplained death and autopsy, August 2000-March 2005, by initial related syndromes
