# Supplementary material for: Unexplained Deaths and Critical Illnesses of Suspected Infectious Cause, Taiwan, 2000–2005
Source: Emerg Infect Dis. 2008 Oct;14(10):1653–5. doi: 10.3201/eid1410.061587 (PMC2609874; doi:10.3201/eid1410.061587)
Supplement: Appendix Table — Frequency distributions of identified pathogens for acute cases with unexplained death and autopsy, August 2000-March 2005, by initial related syndromes [file 06-1587_appT-s1.pdf]

Appendix Table. Frequency distributions of identified pathogens for acute cases with unexplained death and autopsy, August 2000–March 2005, by initial related syndromes

Syndrome,* cause	Acute cases, no. deaths/autopsies								
	Total	Neurologic	Respiratory	Hemorrhagic	Diarrhea	Jaundice	Dermatologic	Heart related	Kidney related
Total†	95/47	24/10	62/32	8/2	12/5	8/3	3/0	8/7	11/7
Infectious causes‡	59/31	17/7	38/22	5/1	9/4	6/3	2/0	6/6	6/4
Bacteria§	31/17	8/3	18/11	5/1	7/3	4/3	2/0	2/2	3/2
Streptococcus spp.	9/4	2/1	3/0	2/0	5/2	1/0	0/0	1/1	1/1
Neisseria meningitidis	4/2	2/0	2/2	3/1	0/0	0/0	1/0	0/0	0/0
Mycoplasma pneumoniae	4/3	1/0	3/3	0/0	0/0	1/1	0/0	1/1	0/0
Pseudomonas aeruginosa	3/1	0/0	3/1	0/0	0/0	0/0	0/0	0/0	1/1
Leptospira interrogans	3/3	2/2	3/3	0/0	0/0	1/1	0/0	0/0	0/0
Other bacteria¶	5/1	1/0	3/1	0/0	1/0	0/0	1/0	0/0	1/0
Unknown bacteria	3/2	0/0	1/0	0/0	1/1	1/1	0/0	0/0	0/0
Virus#	22/12	8/3	15/9	0/0	1/0	1/0	0/0	3/3	3/2
SARS coronavirus**	6/3	0/0	6/3	0/0	0/0	0/0	0/0	0/0	1/1
Enterovirus	5/2	3/0	1/1	0/0	1/0	0/0	0/0	2/2	1/1
Influenza virus	4/2	2/1	4/2	0/0	0/0	0/0	0/0	0/0	0/0
Hepatitis virus	2/1	0/0	0/0	0/0	0/0	1/0	0/0	0/0	1/0
Rabies virus	1/1	1/1	0/0	0/0	0/0	0/0	0/0	1/1	0/0
Hantavirus	1/1	0/0	1/1	0/0	0/0	0/0	0/0	0/0	0/0
Unknown virus	3/2	2/1	3/2	0/0	0/0	0/0	0/0	0/0	0/0
Bacteria and virus††	1/1	0/0	1/1	0/0	1/1	0/0	0/0	0/0	0/0
Other microorganisms	5/1	1/1	4/1	0/0	0/0	1/0	0/0	0/0	0/0
Noninfectious cause	14/8	3/1	8/5	1/0	0/0	1/0	0/0	0/0	2/1
Unclassified	22/8	4/2	16/5	2/1	3/1	1/0	1/0	2/1	3/2

*There were 2 infectious cases and 2 noninfectious cases that could not be categorized into any of these 8 syndromes.
†All infectious agents were identified according to the standard operating procedures for laboratory tests published by the Taiwan Centers for Disease Control (available from http://www.cdc.gov.tw).
‡Autopsies were performed for 47 of the 95 fatal cases; 31 of these were determined to have infectious causes. Among these 31 cases, 7 cases were diagnosed only by autopsy. The other 24 cases were verified by additional laboratory tests: 6 by DNA identification, 8 by serologic test, 12 by culture (1 verifiedoth by bserologic test and culture, 1 by both DNA identification and culture).
§For bacterial pathogens, the organism-specific diagnosis is made only if microbiologic culture provides valid evidence.
¶The etiologic agents included *Burkholderia pseudomallei, Hemophilus bacillus, Mycobacterium tuberculosis, Klebsiella pneumoniae, Staphyloccus aureus*; each of them caused only 1 case.
#For viral pathogens, 2 serial serologic tests must be present for making a diagnosis. If there is only 1 specimen available, at least positive immunoglobulin (Ig) G and IgM either by enzyme immunosorbent assay or indirect immunofluorescence assay, or both, are necessary. Additional testing included nucleic acid amplification by PCR for selected viral pathogens if adequate specimens could be found.
**SARS, severe acute respiratory syndrome.
††Only 1 case with multiple infections including *Pseudomonas aeruginosa, K. pneumoniae,* and Adenovirus.
